# Combination Radioimmunotherapy Approaches and Quantification of Immuno-PET

**DOI:** 10.1007/s13139-015-0392-7

**Published:** 2016-01-26

**Authors:** Jin Su Kim

**Affiliations:** Molecular Imaging Research Center, Korea Institute of Radiological and Medical Sciences, 75 Nowon-Gil, Gongneung-Dong, Nowon-Gu, Seoul, 01812 Korea; Korea Drug Development Platform using Radio-Isotope(KDePRI), Seoul, Korea; Radiologcial and Medico-Oncological Sciences, University of Science and Technology (UST), Seoul, Korea

**Keywords:** Monoclonal antibody, Radioimmunotherapy, Immuno-PET, Tumor microenvironment

## Abstract

Monoclonal antibodies (mAbs), which play a prominent role in cancer therapy, can interact with specific antigens on cancer cells, thereby enhancing the patient’s immune response via various mechanisms, or mAbs can act against cell growth factors and, thereby, arrest the proliferation of tumor cells. Radionuclide-labeled mAbs, which are used in radioimmunotherapy (RIT), are effective for cancer treatment because tumor associated-mAbs linked to cytotoxic radionuclides can selectively bind to tumor antigens and release targeted cytotoxic radiation. Immunological positron emission tomography (immuno-PET), which is the combination of PET with mAb, is an attractive option for improving tumor detection and mAb quantification. However, RIT remains a challenge because of the limited delivery of mAb into tumors. The transport and uptake of mAb into tumors is slow and heterogeneous. The tumor microenvironment contributed to the limited delivery of the mAb. During the delivery process of mAb to tumor, mechanical drug resistance such as collagen distribution or physiological drug resistance such as high intestinal pressure or absence of lymphatic vessel would be the limited factor of mAb delivery to the tumor at a potentially lethal mAb concentration. When α-emitter-labeled mAbs were used, deeper penetration of α-emitter-labeled mAb inside tumors was more important because of the short range of the α emitter. Therefore, combination therapy strategies aimed at improving mAb tumor penetration and accumulation would be beneficial for maximizing their therapeutic efficacy against solid tumors.

## Introduction

Monoclonal antibodies (mAbs), which play a prominent role in cancer therapy, can interact with specific antigens on cancer cells, thereby enhancing the patient’s immune response via various mechanisms, or mAbs can act against cell growth factors and, thereby, arrest the proliferation of tumor cells. Radionuclide-labeled mAbs, which are used in radioimmunotherapy (RIT), are effective for cancer treatment because tumor associated-mAbs linked to cytotoxic radionuclides can selectively bind to tumor antigens and release targeted cytotoxic radiation [1–3]. Iodine-131 (^131^I)-labeled mAbs has been widely used in the successful treatment of patients with lymphoma in nuclear medicine [4–6]. Recently, α-emitter-labeled RIT is considered a promising therapeutic strategy, because α emitters provide high linear energy transfer to tumors within a short range [7].

Although the concept of RIT may appear straightforward, in practice it has been difficult to achieve substantial clinical success, particularly in solid tumors due to the limited delivery of mAb into tumor [8, 9]. Furthermore, limited delivery of mAb would be more problematic in therapy when α-emitter-labeled RIT was performed because of the short range of α particles. In this paper, delivery strategies for mAb in RIT, and the imaging characteristics of some currently and widely used radioisotopes such as iodine-124 (^124^I), zirconium-89 (^89^Zr), and copper-64 (^64^Cu) are discussed. Macro-level and microcellular-level dosimetry strategies are also described.

### Immuno-PET

Immunological positron emission tomography (immuno-PET), which is the combination of PET with mAb, is an attractive option for improving tumor detection and mAb quantification. Immuno-PET has advantages over conventional radioimmunoscintigraphy in terms of accurate quantification of the mAb from the images [10, 11]. The radioisotopes most often used for immuno-PET are: ^64^Cu (half-life [t_1/2_], 12.7 h), and ^124^I (t_1/2_, 4.2 days) [12]. Although ^64^Cu was widely used for the clinical imaging of mAb [13, 14], t_1/2_ of ^64^Cu is too short to prove effective with the slow pharmacokinetic profile of mAb in humans. The accumulation of mAb in tumors is a slow process, as is their clearance from the blood (t_1/2_, 50–90 h) [10]. Slow process of mAb delivery to tumor was due to limited transport of mAb, which was described in the “limited transport of mAb to tumor” section in detail. Table 1 shows the physical characteristics of ^89^Zr, ^124^I and ^18^F. PET radionuclides with a relatively long t_1/2_, such as ^124^I (t_1/2_, 4.2 days) and ^89^Zr (t_1/2_, 78.41 h), would be more suitable for immuno-PET than those with a short t_1/2_.

**Table 1:** Physical characteristics of ^89^Zr, ^124^I and ^18^F

Properties	^89^Zr	^124^I	^18^F
Half-life	78.4 h	4.18 day	109.8 min
Mean β^+^ energy	0.40 MeV	0.83 MeV	0.25 MeV
Mean β^+^ range in water	1.23 mm	3.48 mm	0.62 mm
Single γ energy	909 keV (99.9 %) 1,657 keV (0.1 %) 1,713 keV (0.8 %)	602 keV (61 %) 723 keV (10 %) 1,691 keV (11 %)	
β^+^ branching ratio	23 %	23 %	97 %

^124^I has been used for immuno-PET, but its imaging characteristics are limited in terms of spatial resolution and image quality. In addition, in vivo dehalogenation of ^124^I is the main limiting factor [15]. The spatial resolution of ^124^I is poorer than that of ^18^F, because it has a longer β^+^ range than that of ^18^F (β^+^ range in water, 3.8 and 0.66 mm for ^124^I and ^18^F, respectively). Jin Su Kim’s group in Korea Institute of Radiological and Medical Sciences (KIRAMS) reported that the spatial resolution of ^124^I was reduced by 19 % compared with that of ^18^F on the ECAT HR+ scanner. The PET image quality with this radionuclide is poor owing to the cascade of γ photons and low β^+^ branching ratio (β^+^ branching ratio of ^124^I, 23 %) [16, 17]. High-energy γ photons (602, 723, and 1691 keV) are emitted in a cascade with the β^+^ The major interference is caused by γ photons with energy levels of 602 keV because their energy level falls within the standard energy window of most PET scanners, which will detect these photons as additional background noise or interference. In addition, the 602-keV γ photons have a high branching ratio of approximately 61 % and, as a result, the dead time of the system is also increased, while its count-rate performance is reduced [16]. Cascade γ photons contribute to background image noise in ^124^I-PET and, therefore, this background interference must be corrected to improve the image quality. Moreover, Preylowski et al. [16] reported that the image quality could be improved after correcting the background noise caused by the higher energy γ photons (602 and 732 keV). The correction of higher energy of γ photons referred to as “prompt γ correction” in the study by Preylowski et al. [16], was implemented in the Siemens Biograph mCT scanner. Preylowski et al.’s algorithm calculated the distribution of prompt coincidence using the convolution prompt γ kernel with attenuation correction, and for random coincidences it also corrects acquired data. Kim’s group developed a prompt γ correction method for ^124^I-PET in a sinogram space [18]. The fraction of prompt γ was derived using comparison of β^+^ branching ratio corrected sensitivity. Briefly, the difference between branching ratio corrected sensitivity of ^124^I and that of ^18^F was a fraction of prompt γ [18]. According to the result, the single γ fraction was 3 % for 350-550 keV, 24 % for 350-650 keV, and 31 % for 350-750 keV. The higher single γ fraction for a wider energy window was due to greater inclusion of 602-keV γ within the energy window. Therefore, background noise count due to higher energy of γ photons would differ from energy window width. According to the Jin Su Kim’s method, “scatter distribution × single γ photon fraction” is the portion of background count due to higher single γ photon which could be calculated using the obtained scatter sinogram and measured branching ratio corrected sensitivity [18]. Recently, Jin Su Kim’s group compared the effect of different filter and reconstruction methods for ^124^I quantification on Siemens Inveon PET scanner [19, 20].

**Fig. 1:**
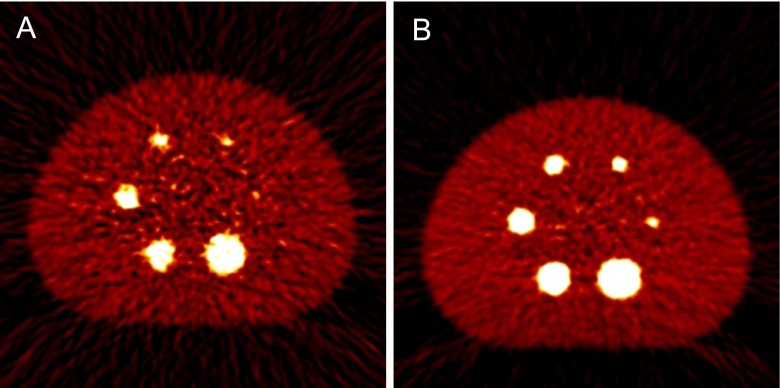
Transaxial images of NEMA IEC phantom using (**a**) ^89^Zr images and (**b**) ^18^F. Images were reconstructed using FBP. This figure is reprinted from Lee et al. [24]

^89^Zr (t_1/2_, 78.41 h) is also an ideal radioisotope for immuno-PET [21–23]. However, ^89^Zr has also poor imaging characteristics because of low spatial resolution and image quality. Jin Su Kim’s group in KIRAMS, Korea, reported that the spatial resolution of ^89^Zr was approximately 9 % lower than that of ^18^F was using the Siemens Biograph Truepoint TrueV scanner (4.5 and 4.1 mm for ^89^Zr and ^18^F, respectively) [24]. The predicted spatial resolution of ^124^I was 5.5 mm on the Siemens Biograph Truepoint TrueV scanner [24]. The value of the β^+^ range was 1.23, 0.62, and 1.23 mm for ^89^Zr, ^18^F, and ^124^I, respectively. The low spatial resolutions of ^89^Zr and ^124^I were due to their long β^+^ ranges. The degradation of the spatial resolution was also observed with animal dedicated PET scanners. According to the report by Disselhorst’s group, the spatial resolution of ^89^Zr was degraded by 10 % compared with that of ^18^F on the Siemens Inveon PET scanner (1.99 and 1.81 mm for ^89^Zr and ^18^F, respectively) [25]. The β^+^ branching ratio of ^89^Zr was 23 %, which is lower than that of ^18^F, but similar to that of ^124^I. Furthermore, the low β^+^ branching ratio of ^89^Zr degraded the image quality compared with that of ^18^F [24]. Figure 1 shows the transaxial images of the National Electrical Manufacturers Association (NEMA) IEC phantom for the comparison of image quality between ^89^Zr and ^18^F. In addition, ^89^Zr emitted 909 keV of γ photons, which falls outside the energy window of clinical PET scanners, but the associated higher energy is still likely to cause Compton scattering. Furthermore, there was the possibility that the scattered photons would be included within the energy window, and produce some degree of noise [24]. Another problem associated with the 909-keV energy of γ photons was the radiation hazard, which was crucial for internal dosimetry and required the necessary provision of radiation protection for both patients and workers. Although there were limitations such as poor spatial resolution and image quality of the immuno-PET when ^89^Zr or ^124^I was used, they were a suitable match for the t_1/2_ of the mAb and, therefore, could provide a tool for monitoring the targeting of mAb to tumors.

### Radioimmunotherapy

RIT such as those using ^131^I tositumomab (Bexxar; GlaxoSmithKline, Philadelphia,PA, USA) [26–28], ^90^Y ibritumomabtiuxetan (Zevalin; Biogen Idec, Cambridge, MA, USA) [29, 30] and ^131^I rituximab (Rituxan; Genentech, South San Francisco, CA, USA) were used for the treatment of hematological tumors. Recently, ^131^I tositumomab was withdrawn for commercial reasons [31]. Regarding RIT for solid tumors, trastuzumab (Herceptin; F. Hoffmann-La Roche, Basel, Switzerland), cetuximab (Erbitux; ImClone Systems, New York, USA), bevacizumab (Avastin; Genentech, South San Francisco, CA, USA) and panitumumab (Vectibix; Amgen, Taos, NM, USA) were used for the treatment.

For the RIT, lutetium-177 (^177^Lu) (t_1/2_, 6.7 days; E_max_, 497 keV; range_max_ in water, 2.1 mm), yttrium-90 (^90^Y) (t_1/2_, 2.7 days; E_max_, 2,270 keV; range_max_ in water, 12.0 mm), and ^131^I (t_1/2_, 8 days, E_max_, 606 keV, range_max_ in water, 2.4 mm) were used. ^90^Y is a pure β emitter, which requires a γ-emitting surrogate (^111^In) for clinical imaging [32]. Glatting et al. [33] and Lhommel et al. [34] reported the feasibility of ^90^Y as a PET radionuclide. However, because, the abundance of β^+^ level of ^90^Y was extremely low (0.003 %), and Bremsstrahlung photons were emitted during the PET acquisition [35], PET imaging with ^90^Y was possibly due to inclusion of Bremsstrahlung photons during PET acquisition. The ^90^Y particle has higher energy and longer particle range, leading to more radioactivity in the tumor cell per peptide molecule and superior crossfire through the tumor, which is especially advantageous in large tumors and tumors with heterogeneous receptor distribution. Furthermore, ^177^Lu particles have lower energy and smaller particle range than ^90^Y does, leading to better absorption in small tumors [32, 36, 37]. Frost et al. [32] showed that the mean absorbed dose of ^177^Lu by the tumor was more than twofold higher than that for ^90^Y following the administration of the same level of radioactivity.

The use of auger electrons [38–40] or α particles was feasible owing to the high linear energy transfer radiation within a short range. Alpha particles such as astatine-211 (^211^At) (t_1/2_, 7.2 h), bismuth-213 (^213^Bi) (t_1/2_, 46 min), actinium-225 (^225^Ac) (t_1/2_, 10 days), bismuth-212 (^212^Bi) (t_1/2_, 60 min), radium-233 (^233^Ra) (t_1/2_, 11 days), terbium-149 (^149^Tb) (t_1/2_, 4 h), and fermium-255 (^255^Fm) (t_1/2_, 20 h) [41–45] would be possible for RIT. ^211^At is of particular interest because of its high linear energy transfer (5,869 and 7,450 MeV α particles), biologically relevant t_1/2_, and absence of α-emitting daughters. Auger electron emitting radionuclides that could be used as theranostic agents are gallium-67 (^67^Ga), bromine-80m (^80m^Br), ^89^Zr, niobium-90 (^90^Nb), technetium-99m (^99m^Tc), indium-111 (^111^In), tin-117m (^117m^Sn), antimony-119 (^119^Sb), iodine-123 (^123^I), iodine-125 (^125^I), platinum-195m (^195m^Pt), and thallium-201 (^201^Tl).

Intranuclear delivery of Auger electron-emitting constructs results in relative biological efficacy similar to that of α emitters but with a comparatively reduced crossfire effect, making them more suitable for single-cell irradiation than the α emitters. Auger electron emitters such as ^125^I have a high linear energy transfer and short range of emission (<10 μm), making them suitable for treating micrometastases while sparing normal tissues [38–40, 46, 47]. In addition, the radiation emitted during the nuclear decay can be used for imaging either using SPECT with γ rays or Bremsstrahlung photons or PET, thereby rendering Auger-electron-emitting radionuclides as ideal theragnosis agents [48, 49]. Kiess’s group at John’s Hopkins University, used a highly specific small molecule targeting the prostate-specific membrane antigen to deliver ^125^I to prostate cancer cells [46]. Hasegawa’s group at the National Institute of Radiological Science, Japan, reported that ^111^In trastuzumab modified with nuclear-localizing signal peptides efficiently delivered an Auger electron emitter ^111^In into the cell nucleus and, therefore, is a promising radiopharmaceutical in Auger electron RIT suitable for targeted killing of HER2-positive cancer cells [39].

Falzone’s group at the University of Oxford, UK evaluated the characteristics of Auger electrons in terms of S values, the effect of cellular geometry and eccentric cell or nucleus arrangements on S values, dose-point kernels (DPKs), and energy deposition on a DNA scale compared with an α emitter, ^223^Ra. The DPK of ^223^Ra and the Auger electron emitters showed that with respect to the energy deposited in spheres of DNA dimensions, only the higher-mass-number Auger electron emitters deposited comparable amounts of energy. However, compared with a mono-energetic 5.77-MeV α particle, the major advantage of Auger electron emitters is that they have a range of less than 11 nm [48, 50].

### Macro-Level and Microcellular-Level Dosimetry

**Fig. 2:**
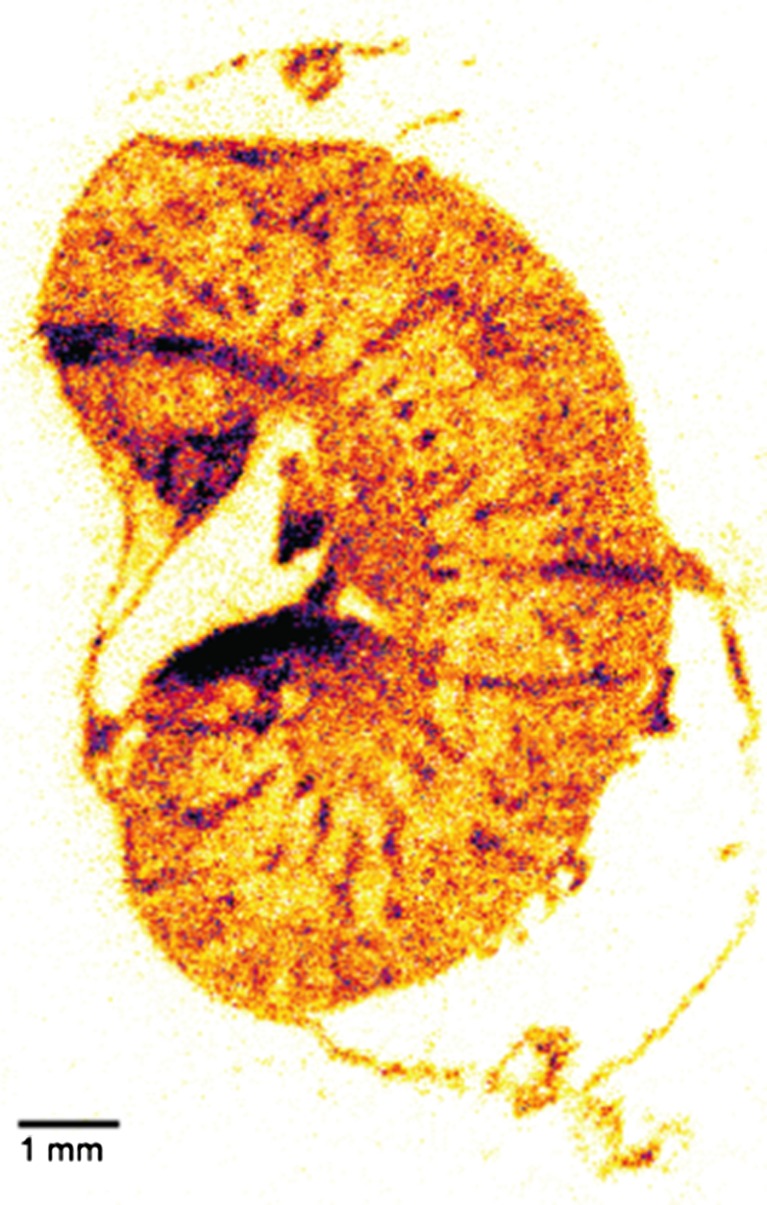
^211^At-1F5 (anti-CD20 mAb) micro-biodistribution for the mouse which was acquired using an α camera. This figure was reprinted from Miller et al. [56]

The absorption of energy from ionizing radiation can cause damage to living tissues, and this effect is used as an advantage in radionuclide therapy. One of the most important factors in the assessment of the effects of radiation on an organ is the amount of radiation energy deposited in that organ. The calculation of radiation energy deposited by internal radionuclides is the focus of internal radiation dosimetry. Currently, whole-body dosimetry was used for RIT, and has proven to be a reliable method for determining the patient-specific maximally tolerated therapeutic radiation dose required to maximize efficacy while minimizing organ and bone marrow toxicity [51, 52]. The Medical Internal Radiation Dosimetry (MIRD) formulation assumed that radioactivity and energy was uniformly distributed and deposited within each organ. This assumption can cause significant errors in the calculated dose as a result of nonpenetrating radiation (e.g., Auger electrons) when the activity was taken up in specific regions or cell types within an organ. Therefore, local radionuclide concentrations and the subsequently absorbed dose can be much higher than average calculations of quantities absorbed by organs might suggest [51]. Dewaraja et al. [53] developed the patient-specific, three-dimensional (3D) method for absorbed dose calculation. Although the mean dose estimates are quite adequate for diagnostic applications, greater accuracy is required for therapeutic applications and patient-specific 3D calculations. With the 3D dosimetry, the minimum and maximum doses, as well as dose nonuniformity can be estimated. Jin Su Kim’s group in KIRAMS, Korea, performed imaging and therapy using ^131^I trastuzumab and a pinhole collimator attached to a conventional gamma camera for human use in a mouse model. Mouse dosimetry and prediction of human dosimetry could be used to provide data for the safety and efficacy of newly developed therapeutic schemes [54]. Meredith et al. [55] reported the first use of dosimetry in human α emitter labeled RIT with ^212^Pb TCMC-trastuzumab. Tools are needed to visualize and quantify the radioactivity distribution and absorbed doses to targeted and non-targeted cells for accurate dosimetry when α-emitter-labeled RIT was performed [56]. Recently, a single-particle digital autoradiography named α camera was developed for the quantification of α particle for targeted radionuclide therapy. Miller et al. [56, 57] developed an α camera called the iQID camera, which is a scintillator-based radiation detection system that images and identifies α particle emissions spatially and temporally using CCD-CMOS cameras and high-performance computing hardware. The iQID camera technique was used for the quantitative imaging of ^211^At activity distributions in cryosections of mouse and dog tissue samples [56]. Figure 2. shows the distribution of ^211^At 1F5 (anti CD20 mAb) in the mouse kidney. Frost et al. [58] reported the result of using ^211^At localization and small-scale dosimetry for optimizing the mAb dose for ^211^At RIT using α-imaging systems.

### Limited Transport of mAb into Tumor

RIT remains a challenge because of the limited delivery of mAb into tumors. The transport and uptake of mAb into tumors is slow and heterogeneous [59]. Although, a large amount of mAb intravenously injected, very little reaches the tumor and a large portion remains circulating in the plasma or is taken up even in normal tissues, thereby irradiating and killing healthy cells over time [60]. Uptaken and penetrated mAb was not evenly distributed within the tissue. Monoclonal antibodies bind to their targets much faster than they diffuse through tissue [61]. Only cells adjacent to blood vessels was more targeted even in well-vascularized regions [62]. The steps associated with targeting mAb to tumors are blood flow to the tumor, extravasation across the blood vessel wall, diffusion into the tissue, and binding to the target [61]. Blood flow occurs in the order of minutes, while binding is completed in several seconds. While the rates of these processes can significantly affect the distribution of the mAb in the tumor, but they do not all have a major impact on the total amount localized in the tissue [61]. Extravasation across the blood vessel wall is the rate-limiting step in uptake. Monoclonal antibodies are transported into tumors by convection and diffusion. Convection of materials in fluids such as mAb in a tumor occurs by the movement of that liquid. This flow occurs when there is a difference in pressure, which forces the fluid from an area of high pressure to that of low pressure. Diffusion occurs as a result of the random Brownian motion of molecules in a liquid. The distance of mAb diffusion is proportional to the square root of time [59, 62–64].

The time required to deliver mAbs to the tumor vasculature can be calculated by measuring the blood flow. The time for blood flow can be calculated as follows

$$ \mathrm{Blood}\ \mathrm{flow}\ \mathrm{t}\mathrm{ime}=1/Q\left(1\ \hbox{--}\ \mathrm{H}\mathrm{t}\right) $$

where *Q* is the flow rate of the blood in the tumor (volume of whole blood per volume of tumor per time), and *Ht* is the hematocrit. The extravasation rate of mAb is very slow compared with measured flow rates and, therefore, the permeability typically has a greater impact on the uptake [60].

The time it takes for the mAbs to penetrate a specified volume of a tumor is calculated as:

$$ \mathrm{Extravasation}\ \mathrm{time}=V/PS $$

where *P* is the permeability and *S/V* is the blood vessel surface area to tumor volume ratio [60].

Convection within the tissue are small compared with the rate of diffusion and, therefore, diffusive movement mainly drives the interstitial transport. Using Fick’s law, diffusion time is calculated as:

$$ \mathrm{D}\approx {\mathrm{R}}^2/\mathrm{D}\varepsilon $$

where *R* is the distance the mAb must diffuse, *D* is the diffusion coefficient between the cells, and *ε* is the void fraction [60].

Extravasation and diffusion were limiting step for delivery of mAbs. According to the mathematical analysis, estimated permeability in step of extravasation was 0.003 μm/s for mAb and 1 μm/s for FDG and diffusion rate was 10 μm^2^/s for mAb and 500 μm^2^/s for FDG [64]. The estimated time-based mathematical analysis for delivering the mAb to the tumor was 10 min for convection, 18 h for extravasation, 24 min for diffusion, and 12 s for binding [59, 60, 64].

### Enhancement of mAb Penetration into Tumor

The tumor microenvironment contributed to the limited delivery of the mAb [65]. The limited targeting and insufficient dose delivery of mAb to solid tumor were caused by abnormal structure of tumor vessel, highly fibrotic or desmoplastic tumor, absence of functional lymphatics, and high fluid permeability [59, 66–68]. Tumor cells are surrounded by layers of extracellular matrix (ECM) proteins (e.g., collagen, elastin, fibronectin, and laminin), which largely prevents the tumor vasculature from penetrating the tumor nests. Tumor-derived ECM plays an important role in inhibiting the penetration and dispersion of cancer therapeutic agents within tumor masses and has been implicated in the resistance of solid tumors to therapy [69]. Beyer et al. [69] observed extensive tumor ECM and intercellular junctions in patients with breast cancer and in xenograft models [70].

Targeting tumors with mAb-based therapeutics is a complex task that presents multiple kinetic barriers. Monoclonal antibody internalization and clearance inhibit uptake both in solid tumors limited by tumor vascular permeability and in micrometastases limited by diffusion [61, 69]. To improve the efficacy of RIT, binding-site barriers need to be surmounted to enhance the distribution of mAb uniformly in tumors. The binding-site barriers can cause non-uniform distribution of mAb in the tumor microenvironment because radiolabeled mAbs bind primarily to the tumor cells nearest to the vasculature. This hinders the uniform distribution of radiolabeled mAbs throughout the tumor unless the dose of mAbs administered is at a concentration that can saturate all antigens on the tumor cells. Nonuniform microdistribution of mAb leads to a marked difference in individual cell survival across the tumor [71]. Therefore, although RIT was shown to be effective against hematological tumors, solid tumors were less responsive due to insufficient dose delivery and radiation resistance [72]. Numerous solutions such as fractionated dosing [73] and mAb pretargeting methods [74], as well as recombinant immunotoxins [75], were introduced in attempts to improve the efficacy of RIT against solid tumors. Yun’s group at Hanynag University, Korea used ECM-degrading oncolytic adenovirus to achieve a desirable therapeutic outcome in pancreatic cancer [76]. Decorin modulates tumor ECM production and, therefore, has an integral role in the degradation or downregulation of tumor ECM constituents or both. The decorin-based approach would be effective for increasing mAb penetration into solid tumors [69, 77]. Chang H. Paik’s group at NIH, USA used paclitaxel (Taxol) [8] or pulsed high-intensity focused ultrasound to enhance the therapeutic efficacy of RIT against solid tumors [9, 78]. Furthermore, combined RIT strategies could be used to cure solid tumors in patients if the accumulation and penetration of mAb into the tumor microenvironment could be increased, thereby sensitizing more tumor cells to the radiation from the radiolabeled mAb.

Regarding microcellular level imaging of mAb distribution, microcellular level studies by Paik et al. [8, 9] showed the penetration and enhanced accumulation of mAbs in tumors following the modulation of the tumor microenvironment [9, 78]. Although conventional clinical nuclear medicine imaging techniques such as PET and SPECT have been widely used in molecular imaging, they have limited spatial resolution for revealing the intratumoral mAb micro-distribution. Therefore, imaging of fluorescent dye-conjugated mAb distribution would be useful to confirm the micro-distribution of mAbs within tumors.

## Conclusion

The tumor microenvironment may play an important role in solid tumor RIT. When α-emitter-labeled mAbs were used, deeper penetration of α-emitter-labeled mAbs inside tumors was more important because of the short range of the α emitter. Moreover, combination therapy strategies aimed at improving mAb tumor penetration and accumulation are crucial and would be beneficial for maximizing their therapeutic efficacy against solid tumors.
